# Characterization of Mouse Monoclonal Antibodies Against the HA of A(H7N9) Influenza Virus

**DOI:** 10.3390/v11020149

**Published:** 2019-02-11

**Authors:** Mutsumi Ito, Seiya Yamayoshi, Kazushi Murakami, Kenji Saito, Atsuo Motojima, Kazunari Nakaishi, Yoshihiro Kawaoka

**Affiliations:** 1 Division of Virology, Department of Microbiology and Immunology, Institute of Medical Science, University of Tokyo, Minato-ku, Tokyo 108-8639, Japan; ito-mu@ims.u-tokyo.ac.jp; 2 TAUNS Laboratories, Inc., Izunokuni, Shizuoka 410-2325, Japan; k_murakami@tauns.co.jp (K.M.); k_saito@tauns.co.jp (K.S.); a_motojima@tauns.co.jp (A.M.); k_nakaishi@tauns.co.jp (K.N.); 3 Department of Special Pathogens, International Research Center for Infectious Diseases, Institute of Medical Science, University of Tokyo, Minato-ku, Tokyo 108-8639, Japan; 4 Department of Pathobiological Sciences, School of Veterinary Medicine, University of Wisconsin-Madison, Madison, WI 53706, USA

**Keywords:** Influenza A virus, H7N9, HA, mouse monoclonal antibody, Neutralization, Antigenic change

## Abstract

Many cases of human infection with the H7N9 virus have been detected in China since 2013. H7N9 viruses are maintained in chickens and are transmitted to humans at live bird markets. During circulation in birds, H7N9 viruses have accumulated amino acid substitutions in their hemagglutinin (HA), which resulted in an antigenically change in the recent H7N9 viruses. Here, we characterized 46 mouse monoclonal antibodies against the HA of the prototype strain. 16 H7-HA-specific monoclonal antibodies (mAbs) possessed hemagglutination inhibition (HI) and neutralization activities by recognizing the major antigenic site A; four other H7-HA-specific clones also showed HI and neutralizing activities via recognition of the major antigenic sites A and D; seven mAbs that reacted with several HA subtypes and possibly recognized the HA stem partially protected mice from lethal infection with prototype H7N9 virus; and the remaining 19 mAbs had neither HI nor neutralization activity. All human H7N9 viruses tested showed a similar neutralization sensitivity to the first group of 16 mAbs, whereas human H7N9 viruses isolated in 2016–2017 were not neutralized by a second group of 4 mAbs. These results suggest that amino acid substitutions at the epitope of the second mAb group appear to be involved in the antigenic drift of the H7N9 viruses. Further analysis is required to fully understand the antigenic change in H7N9 viruses.

## 1. Introduction

The first severe human cases of influenza A(H7N9) virus infection were reported in the spring of 2003 [1]. Phylogeny told us that these viruses originated from a reassortment among different avian influenza viruses [2]; the hemagglutinin (HA) and neuraminidase (NA) segments were derived from H7N3 viruses and N9 viruses, respectively, and the PB2, PB1, PA, NP, M, and NS segments were derived from H9N2 viruses [3,4,5]. The H7N9 virus has continued to infect humans every influenza season, mainly in China, with the fifth wave occurring in the 2016–17 season [6] and a limited number of human cases being reported in the 2017–18 season. In particular, the Yangtze River Delta and Pearl River Delta regions saw large numbers of H7N9 cases in the 2016–17 season [6]. As of 13 December 2018, a total of 1567 laboratory-confirmed human cases and at least 615 related deaths have been reported (https://www.who.int/influenza/human_animal_interface/HAI_Risk_Assessment/en/). During the fifth wave, highly pathogenic H7N9 viruses possessing HA with multi-basic amino acids at the cleavage site were isolated from avian and human cases [7,8]. The highly pathogenic H7N9 viruses that were isolated from these human cases possessed amino acid mutations in HA and NA that enhances binding to human-type receptors and are associated with resistance to NA inhibitors [8,9]. One of these isolates, A/Guangdong/17SF003/2016, possessed three amino acid changes, two in PB2 and one in PA, that enhanced virus polymerase activity, virus growth in cultured cells, and pathogenicity in mice [10]; this virus also transmitted among ferrets via respiratory droplets [11]. The emergence of such highly pathogenic H7N9 viruses is a serious threat to public health.

During circulation in avian species, H7N9 viruses accumulate amino acid mutations in HA that may affect the antigenicity of HA. Recently, antisera from infected ferrets revealed that the antigenicity of some human H7N9 viruses isolated during the fifth wave in the 2016–2017 season differed from that of human H7N9 viruses isolated in 2013 [8,12]. Although the HA-L226Q (H3 numbering) or HA-S128N+A135T/S mutation affects the antigenicity of H7N9 virus and some H7N9 viruses that were isolated in 2017 possess these mutations [13,14], the amino acid substitutions responsible for the antigenic changes of isolates obtained from humans in the 2016–2017 influenza season were not fully understood until now [15]. Meanwhile, several monoclonal antibodies (mAbs) against H7-HA have been obtained to characterize virus antigenicity or for clinical use [16,17,18,19,20,21]. Although the epitopes of several mAbs have been determined, their reactivity against H7N9 viruses isolated in 2016–2017 has not yet been investigated and mAb availability has been limited. Therefore, additional mAbs are needed to characterize the antigenic change in H7N9 viruses isolated in 2016–2017.

We previously reported the generation of 46 mouse mAbs against the HA of human H7N9 virus, which was isolated in 2013 [22] and utilized some of them for the development of a rapid diagnostic test specific for H7-subtype viruses and the antigenic characterization of feline H7N2 viruses [22,23]. Here, we characterized all 46 mouse mAbs by using various virologic assessments.

## 2. Materials and Methods

### 2.1. Ethics and Biosafety Statements

The research protocol for the experiments with mice for mAb production was approved by and is in accordance with the policies and procedures of Tauns laboratories, Shizuoka, Japan. All experiments with mice for in vivo protection were performed in accordance with the University of Tokyo’s Regulations for Animal Care and Use and were approved by the Animal Experiment Committee of the Institute of Medical Science, the University of Tokyo.

All experiments with H7N9 viruses were performed in biosafety level 3 (BSL3) laboratories at the University of Tokyo, which are approved for such use by the Ministry of Agriculture, Forestry, and Fisheries, Japan.

### 2.2. Cells

Madin-Darby canine kidney (MDCK) cells were maintained in Eagle’s minimal essential medium (MEM) containing 5% newborn calf serum (NCS). Human embryonic kidney 293T cells were maintained in Dulbecco’s modified Eagle’s medium (DMEM) containing 10% fetal calf serum (FCS). These cells were incubated at 37 °C under 5% CO_2_.

### 2.3. Viruses

The clinical isolate A/Anhui/1/2013 (Anhui/1; H7N9), which was passaged and propagated in eggs [24], was used for the selection of escape mutants and mouse challenge tests. All wild-type and mutant Anhui/1 viruses and the A/Taiwan/1/2017 (H7N9) virus that were rescued from cloned plasmids [25,26] and propagated in eggs or MDCK cells were used for other experiments. All viruses were titrated in MDCK cells by means of plaque assays.

### 2.4. Hybridomas

The hybridomas used in this study were obtained previously [22]. Mice immunized with inactivated Anhui/1 were used for hybridoma production. The reactivity of secreted mAbs from the hybridoma cell lines was first examined by using an ELISA with purified recombinant HA proteins or purified virions of Anhui/1. The subclass of each clone was determined by using a monoclonal sub-isotyping kit (American Qualex, San Clemente, CA).

### 2.5. Reactivity of Mouse mAbs

96-well microplates coated with each recombinant soluble HA protein with the original amino acid sequence derived from A/California/07/2009 (H1N1pdm09), A/Canada/720/2005 (H2N2), A/Perth/16/2009 (H3N2), A/Indonesia/5/2005 (H5N1), A/Egypt/N05056/2009 (H5N1), A/northern shoveler/California/HKWF115/2007 (H6N1), A/ruddy turnstione/New Jersey/563/2006 (H7N2), A/Netherlands/219/2003 (H7N7), Anhui/1 (H7N9), A/Hong Kong/35820/2009 (H9N2), B/Malaysia/2506/2004 (Victoria-lineage), and B/Florida/4/2006 (Yamagata-lineage), all of which were purchased from Sino Biological, or purified Anhui/1 (H7N9) virus were reacted with each mAb, followed by enhanced chemi luminescence (ECL) Mouse IgG, horseradish peroxidase (HRP)-Linked Whole Ab (GE healthcare, Tokyo, Japan).

### 2.6. Hemaggulitinin Inhibition Assay

Purified antibody (50 µg/mL) was serially two-fold diluted with PBS prior to being mixed with 8 HA units of Anhui/1. Antibody-virus mixtures were incubated for 60 min at room temperature and then mixed with 0.5% chicken red blood cells. After a 60-min incubation at room temperature, hemagglutination was assessed. The minimum mAb concentration for hemagglutinin inhibition was expressed as the HI value (μg/mL).

### 2.7. Virus Neutralization Assay

Purified antibody (50 µg/mL) in quadruplicate was serially two-fold diluted with MEM containing 0.3% bovine serum albumin (BSA-MEM) prior to being mixed with 100 or 200 TCID_50_ (50% tissue culture infectious doses) of the indicated viruses at 37 °C for 30 min. The mixtures were inoculated into MDCK cells and incubated for 1 h at 37 °C. BSA-MEM containing N-tosyl-Lphenylalanine chloromethyl ketone (TPCK)-treated trypsin was added to each well and the cells were incubated for three days at 37 °C. The cytopathic effect (CPE) was examined, and antibody titers required to reduce virus replication by 50% (IC_50_) were determined by using the Reed and Muench formula.

### 2.8. Evaluation of the In Vivo Protective Efficacy of the mAbs in Mice

Baseline body weights of six-week-old female BALB/c mice (Japan SLC) were measured. Three mice per group were intraperitoneally injected with the indicated antibodies at a concentration of 15 mg/kg [27,28]. One day later, the mice were anesthetized and challenged with 10 mouse lethal dose 50 (MLD_50_) (50 µL) of Anhui/1. Body weight and survival were monitored daily for 14 days. Mice with body weight loss of more than 25% of their pre-infection values were humanely euthanized.

### 2.9. Generation of Escape Mutant Viruses

10-fold serially diluted Anhui/1 was incubated with each mAb (100 μg/mL) for 30 min at 37 °C. The mixture was inoculated to MDCK cells for 1 h at 37 °C. After removal of the inoculum, infected cells were cultured in BSA-MEM in the presence of trypsin (1 μg/mL) and each mAb (100 μg/mL). At two days after infection, CPE was examined and culture media from CPE-positive wells infected with the highest virus dilution were harvested. The collected culture media were incubated with each mAb (100 μg/mL) for 30 min at 37 °C prior to being subjected to a standard plaque assay. Five plaque-purified viruses per mAb were propagated in MDCK cells. The open reading frame of the HA of the plaque-purified viruses was directly sequenced by Sanger sequencing. Amino acid changes identified in three or more plaque-purified viruses were considered as substitutions that were potentially important for the escape from each mAb.

### 2.10. Phylogenetic Analysis

The phylogenetic tree of the 862 HA nucleotide sequences derived from human H7N9 viruses was constructed by using the neighbor-joining (NJ) method with the Kimura two-parameter method and the bootstrap procedure (*n* = 100) using the MEGA 7.0.26 software. Sequence data were obtained from the GISAID database on 24 April 2018. The sequencing data set used in this study is available upon request.

### 2.11. Virus Rescue

Plasmid-based reverse genetics for virus generation was performed as previously described [29]. RNA polymerase I plasmids encoding the HA gene of A/Huzhou/1/2013 (H7N9), A/Shantou/1001/2014 (H7N9), A/Guangdong/0048/2014 (H7N9), A/Zhejiang/22/2014 (H7N9), A/Anhui/09186/2014 (H7N9), A/Fujian/1/2016 (H7N9), A/Hong Kong/VB16049808/2016 (H7N9), A/Hong Kong/214/2017 (H7N9), A/Hunan/02287/2017 (H7N9), A/Zhejiang/15/2016 (H7N9), A/Zhejiang/6/2017 (H7N9), A/Anhui/60928/2016 (H7N9), or A/Zhejiang/2/2017 (H7N9), the NA gene of Anhui/1 [26] or A/chicken/Huaian/003/2015 (H7N9), and six RNA polymerase I plasmids encoding the other six segments of wild-type or high-yield A/Puerto Rico/8/34 (H1N1) [30] were used. All sequences were synthesized based on the sequences in the GISAID database. Each rescued virus was propagated in MDCK cells and stored as a stock virus. The HA gene of all rescued viruses was sequenced to confirm the absence of unwanted mutations.

### 2.12. Molecular Modeling

The structural model of the H7-HA from A/Shanghai/1/2013 (H7N9) (PDB code, 4LCX) was used to assign the amino acid positions with the PyMOL Molecular Graphics System, version 1.3.

## 3. Results

### 3.1. Reactivity of 46 Mouse Monoclonal Antibodies

A total of 46 hybridomas that produced mouse monoclonal antibodies (mAbs) against H7-HA were generated previously [22]. Although we obtained several mAbs against the virus proteins NP and M1, we focused on the mAbs against HA. To evaluate their breadth of reactivity, we performed an ELISA with all 46 mAbs and recombinant HA proteins of H1, H2, H3, H5, H6, H7, and H9 viruses, as well as B/Yamagata-, and B/Victoria-HA. Seven clones (clones #1 through #7) recognized several subtypes of HA; in particular, clones 14-24-5 (#6) and 21-12-10 (#7) recognized all subtypes of HA tested other than type B-HA (Table 1). The remaining 39 clones (#8 through #46) specifically recognized H7-HA of A/Netherland/219/2003 (H7N7) and A/Anhui/1/2013 (Anhui/1, H7N9) but did not bind to H7-HA derived from A/ruddy turnstione/New Jersey/563/2006 (H7N2) (Table 1). All of the tested mAbs bound to HA on the Anhui/1 virion (Table 1).

Next, we examined whether these 46 mAbs possess hemagglutinin inhibition (HI) activity and virus neutralization activity against Anhui/1 in vitro. Clones #1 through #7 and #28 through #46 showed no HI and no neutralization potency at a concentration of 50 μg/mL, except for clone 11-21-22 (#3), which possessed weak HI activity and no neutralization activity in vitro (Table 2). Clones #8 through #27 inhibited virus hemagglutination and virus infection at 0.39–12.5 and 0.62–8.84 μg/mL, respectively (Table 2). These results together with the cross-reactivity data suggest that clones #1 through #7 recognize the HA stem and clones #8 through #27 target the HA head. Clones #28 through #46 failed to inhibit virus infection in vitro; further analysis is needed to determine whether they play a role in vivo.

### 3.2. In Vivo Protective Efficacy of Cross-Reactive Clones

**Figure 1:**
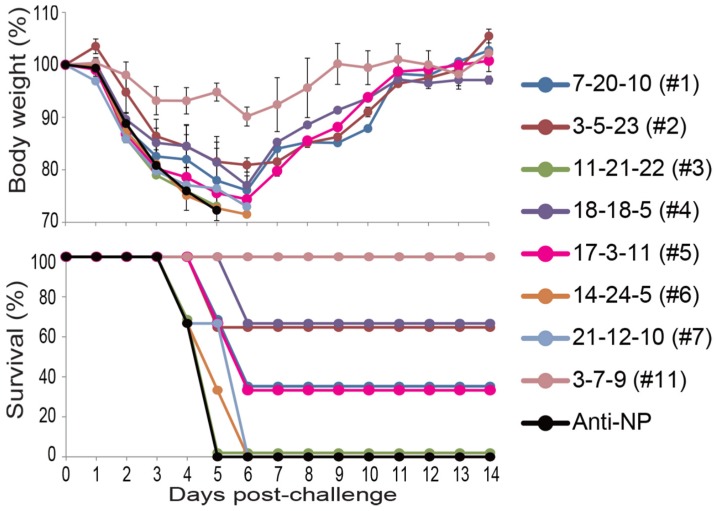
In vivo protective efficacy in mice. Three mice per group were intraperitoneally injected with the indicated antibodies at 15 mg/kg. One day later, the mice were intranasally challenged with 10 mouse lethal dose 50 (MLD_50_) of Anhui/1. Body weight and survival were monitored daily for 14 days. A mouse anti-NP mAb at 15 mg/kg served as a negative control.

We examined the in vivo protective efficacy of clones #1 through #7 because some anti-HA stem antibodies protect mice by activating Fc-mediated effector functions without virus neutralization [27,31,32]. Mice were intranasally challenged with 10 MLD_50_ of Anhui/1 one day after intraperitoneal injection of each clone at a concentration of 15 mg/kg. A mouse mAb against the NP protein of influenza A virus and a neutralizing mAb against the HA head [clone 3-7-9 (#11)] served as negative and positive controls, respectively. All mice that received clone 11-21-22 (#3), 14-24-5 (#6), or 21-12-10 (#7) died within 5–6 days, as did mice that received the anti-NP mAb (Figure 1). Two of the three mice that received clone 3-5-23 (#2) or 18-18-5 (#4) and one of the three mice that received clone 7-20-10 (#1) or 17-3-11 (#5) survived for two weeks after the challenge infection, although all of the mice transiently lost a considerable amount of body weight. Clone 3-7-9 (#11) protected all mice from lethal infection with Anhui/1 with mild body weight loss. These results show that non-neutralizing mAbs have the potential to partially protect mice from H7N9 virus infection independent of the subclass of the mAbs.

### 3.3. Acquisition of Mutant Viruses that Escaped from Neutralizing mAbs

To determine the epitope(s) of the neutralizing mAbs, we attempted to obtain mutant Anhui/1 viruses that escaped from each neutralizing clone. We used the egg-passaged virus for escape mutant selection, because virus diversity should be higher in egg-passaged viruses than viruses generated by reverse genetics, making it easier to obtain escape mutants. Mutant viruses that escaped from clones #8 through #23 possessed the G144E mutation in HA and some of them also possessed the V505A mutation (Table 3). The A135T mutation in HA was found in a mutant virus that escaped from clones #24 through #27 and some other mutations, including L226Q, which was found in three escape mutants, were also detected (Table 3). The G144E mutation in the HA of Anhui/1 allowed escape from clones 3-9-18-7 (#13), 8-10-16 (#14), 8-13-19 (#15), 10-19-19 (#16), and 17-3-7 (#19). These results, together with HI data, suggest that G144E plus V505A and A135T plus L226Q are likely involved in evasion from recognition.

To determine which amino acid mutation plays a central role in evasion, we prepared mutant viruses possessing the G144E+V505A, G144E, A135T+L226Q, A135T, or L226Q substitution in the HA, and tested the neutralization potency of the remaining 15 mAbs against these mutant viruses. The mutant virus possessing HA-G144E+V505A was not neutralized by clones 17-16-16 (#8), 2-20-20 (#9), 3-5-4 (#10), 3-7-9 (#11), 3-7-19 (#12), 11-8-3 (#17), 11-11-2-7 (#18), 17-16-28 (#20), 19-17-20 (#21), 22-8-6 (#22), or 9-15-3 (#23) at 50 μg/mL (Table 4). Since these mAbs likely target the HA head and the V505A mutation is located in the HA stem, we examined the HA-G144E mutant virus for neutralization of representative mAb clones 3-7-19 (#12) and 11-8-3 (#17), and found that both clones failed to neutralize the HA-G144E mutant. Both the HA-G144E/V505A and HA-G144E viruses were neutralized by clones 13-9-19-7 and 19-9-13 at a similar concentration to wild-type virus. The mutant virus possessing HA-A135T/L226Q was not neutralized by clones 10-2-9 (#25), 10-3-17 (#26), or 19-9-13 (#27), whereas the neutralization titer of clone 13-9-19-7 (#24) against this virus was reduced compared with that against wild-type virus (Table 4). Both mutant viruses possessing each single mutation (HA-A135T and HA-L226Q) were similarly neutralized by clones 13-9-19-7 (#24), 10-2-9 (#25), 10-3-17 (#26), and 19-9-13 (#27), except for HA-A135T by clone 19-9-13 (#27). These results suggest that acquisition of the single G144E mutation or the A135T+L226Q double mutation is important for evasion from the tested mAbs. Taken together, our neutralizing mAbs can be roughly classified into two groups based on the escape mutations: clones #8 through #23 and clones #24 through #27.

### 3.4. Neutralization of mAbs Against Human H7N9 Isolates Detected Between 2013 and 2017

**Figure 2:**
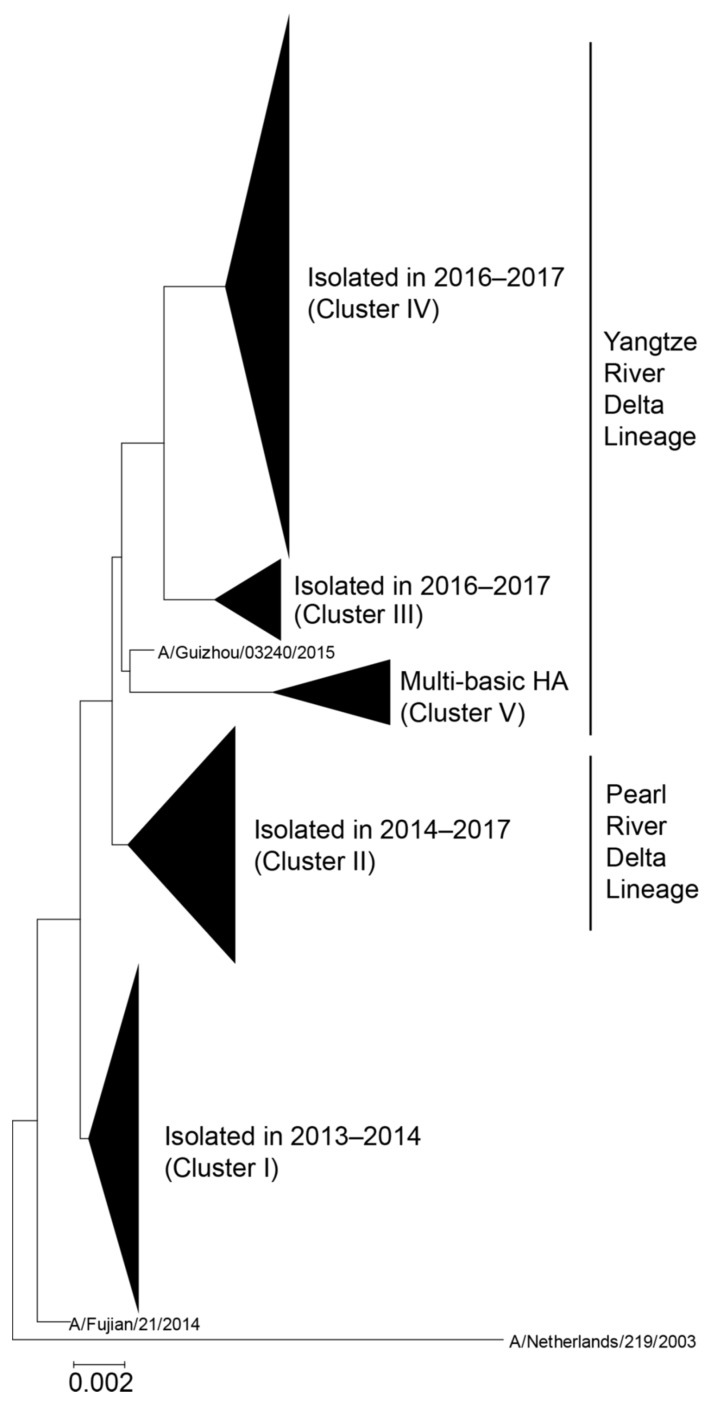
Phylogenetic tree of HA sequences derived from human H7N9 viruses. The evolutionary history was inferred using the Neighbor-Joining method with Kimura distances. Five major clusters are shown as a collapsed branch. A/Netherlands/219/2003 is defined as an outgroup. The Yangtze River Delta and Pearl River Delta lineages are circulating in China. Highly pathogenic H7N9 viruses, which harbor multiple basic amino acids in the HA cleave site, are included in the Yangtze River Delta lineage.

**Figure 3:**
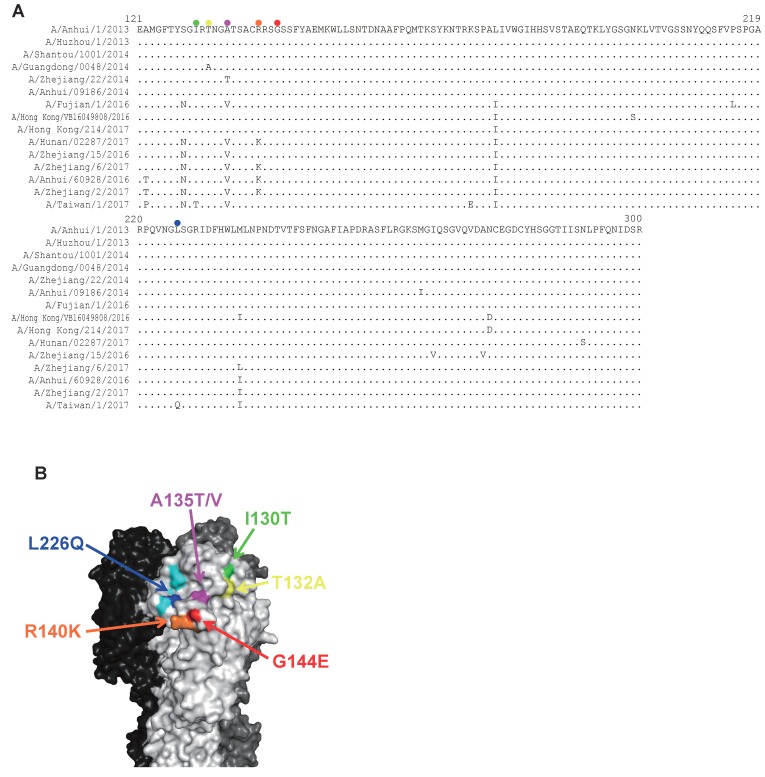
Amino acid substitutions potentially involved in evasion from neutralizing mAbs. (A) Alignment of H7-HA sequences. Amino acid sequences of HA derived from the human H7N9 viruses tested in Table 5 were aligned. Since all tested mAbs targeted the HA head, the HA head sequences are shown. Each colored circle indicates each position on the HA structure. (B) Amino acid substitution sites mapped onto the H7-HA molecule. Amino acid mutations that were identified from escape mutant viruses (A135V, G144E, and L226Q) and substitutions that appear to be important for evasion from mAb recognition (I130T, T132A, A135T, and R140K) were mapped onto the three-dimensional (3D) structure of the H7-HA trimer (PDB; 4LCX) by using the molecular graphics system PyMOL. Cyan indicates the receptor binding site.

Since ferret antisera revealed that the antigenicity of recent H7N9 viruses has changed since 2013 [8], we elucidated the neutralizing capability of our mAbs against representative human H7N9 viruses isolated between 2013 and 2017. To select isolates based on a phylogenetic analysis, we downloaded the 862 HA nucleotide sequences of human H7N9 virus and constructed the phylogenetic tree by using the neighbor-joining (NJ) method with the Kimura two-parameter method. According to the tree, human H7N9 viruses are divided into five clusters (Figure 2): cluster I includes isolates isolated in 2013–2014, cluster II includes isolates of the Pearl River Delta lineage isolated in 2014–2017, clusters III and IV include isolates of the Yangtze River Delta lineage isolated in 2016–2017, and cluster V mainly includes isolates of highly pathogenic H7N9 viruses of the Yangtze River Delta lineage. We selected 15 human isolates as representatives of each cluster for our neutralizing experiments: A/Anhui/1/2013, A/Huzhou/1/2013, A/Shantou/1001/2014, A/Guangdong/0048/2014, A/Zhejiang/22/2014, and A/Anhui/09186/2014 were picked from cluster I, A/Fujian/1/2016, A/Hong Kong/VB16049808/2016, A/Hong Kong/214/2017, A/Hunan/02287/2017, and A/Zhejiang/15/2016 from cluster II, A/Zhejiang/6/2017 from cluster III, A/Anhui/60928/2016 and A/Zhejiang/2/2017 from cluster IV, and A/Taiwan/1/2017 from cluster V (Table 5). We then prepared reassortant viruses possessing each HA above, the NA of Anhui/1 or A/chicken/Huaian/003/2015 (H7N9), and the other six segments of wild-type or high-yield A/Puerto Rico/8/34 (H1N1) [30]. These viruses were evaluated in a neutralization assay using 20 neutralizing mAbs. Most of the recombinant viruses were similarly neutralized by these mAbs, although the neutralization values were higher in several combinations (Table 5). In particular, clones 13-9-19-7 (#24), 10-2-9 (#25), 10-3-17 (#26), and 19-9-13 (#27) tended to show reduced neutralization activity against A/Guangdong/0048/2014, A/Zhejiang/22/2014, A/Hong Kong/214/2017, A/Hunan/02287/2017, A/Zhejiang/15/2016, A/Zhejiang/6/2017, A/Anhui/60928/2016, A/Zhejiang/2/2017, and A/Taiwan/1/2017, suggesting that these nine viruses possessed amino acid substitutions to escape from these four clones. To identify such substitutions, we compared the amino acid sequences of their HA head (Figure 3A). To escape from clones 13-9-19-7 (#24), 10-2-9 (#25), 10-3-17 (#26), and 19-9-13 (#27), the A135T and L226Q mutations were required for Anhui/1 (Table 4). We also looked at amino acid substitutions around these two positions on the HA molecule. A/Guangdong/0048/2014 harbored the T132A substitution (orange) and A/Zhejiang/22/2014 possessed the A135T substitution (purple), which created an additional glycosylation site at positions 133–135 (NGT) (Figure 3B). A/Hunan/02287/2017, A/Zhejiang/6/2017, A/Anhui/60928/2016, and A/Zhejiang/2/2017 possessed the A135V (purple) and R140K (yellow) substitutions. A/Zhejiang/15/2016 harbored the A135V substitution (purple). A/Taiwan/1/2017 possessed the I130T (green), A135V (purple), and L226Q (indigo blue) substitutions. The I130T substitution together with S128N in HA of A/Taiwan/1/2017 created another glycosylation site at positions 128–130 (NGT). A/Hong Kong/214/2017 did not have any substitutions around positions 135 and 226. These results suggest that these amino acid substitutions and creation of a novel N-linked glycosylation site might be involved in the low susceptibility of recent human H7N9 viruses to mAb clones [13-9-19-7 (#24), 10-2-9 (#25), 10-3-17 (#26), and 19-9-13 (#27)].

## 4. Discussion

Here, we characterized 46 mouse anti-HA mAbs and classified them into three groups: 20 H7-HA-specific mAbs possessing HI and neutralization activities, seven mAbs that cross-react with several HA subtypes and possibly recognize the HA stem, and 19 H7-HA-specific mAbs that show no HI or neutralization activity. Epitope mapping using escape mutants and reactivity to 15 human H7N9 viruses revealed 20 neutralizing mAbs that could be roughly divided into two groups. The first group includes clones #8 through #23, which mainly recognize an epitope that includes the amino acid at position 144, which is a part of the major antigenic site A. Mouse monoclonal antibodies 5A6, 4A2, and 2C4 also recognize the antigenic site A [16,33]. The monoclonal antibodies H7.169, 07-5D03, 07-4D05, 07-4B03, and 07-4E02, which were isolated from human volunteers who received H7N9 vaccines, target the amino acids in antigenic site A [18,19]. Thus, our results confirm that the antigenic site A is one of the dominant epitopes in H7-HA. However, the R140K substitution in some human H7N9 isolates, which is also located at antigenic site A, did not affect the neutralization of our mAbs. Therefore, the precise binding mode of our mAbs with H7-HA should be elucidated with further analysis. The second group, which includes clones #24 through #27, recognized an epitope that included the amino acids at positions 135 and 226, which located at major antigenic sites A and D, respectively. Since this group of mAbs failed to neutralize the recent human H7N9 viruses, amino acid substitutions at the epitope of clones #24 through #27 might be involved in the antigenic difference between human isolates obtained in 2013 and those obtained during the 2016–2017 season [8,12]. These results are consistent with previous reports of the A135T and L226Q mutations being responsible for the antigenic change in recent human isolates [13,14].

None of the mAbs that cross-reacted with the HAs of several subtypes had HI activity and inhibited virus replication in vitro, suggesting that they are likely to target the HA stem. Some mAbs against the HA stem without neutralization activity can protect mice via Fcγ receptor-mediated effector cell activation [27,31,32,34,35], such as antibody-dependent cellular cytotoxicity (ADCC) by natural killer cells, antibody-dependent cellular phagocytosis (ADCP) by macrophages, and antibody-dependent neutrophil-mediated phagocytosis (ADNP) by neutrophils [36,37]. Therefore, it may be that our mouse mAbs that are cross-reactive with several HA subtypes also protect mice from lethal challenge infection by inducing such activities.

H7N9 viruses accumulate amino acid substitutions in HA during enzootic transmission among birds. Antigenic characterization by using mAbs is useful to monitor the antigenic drift of H7N9 viruses. Our H7-HA-specific mAbs that recognize epitopes around the amino acid at position 144 or that include the amino acids at positions 135 and 226 would be useful for such purpose.

## Figures and Tables

**Table 1:** Reactivity of monoclonal antibodies (mAbs) against the recombinant hemagglutinin (HA).

#	Clone	Subclass	H1 ^a^	H2 ^b^	H3 ^c^	H5-1 ^d^	H5-2 ^e^	H6 ^f^	H7-1 ^g^	H7-2 ^h^	H7-3 ^i^	H9 ^j^	B-1 ^k^	B-2 ^l^	H7N9 Virus ^m^
**1**	7-20-10	IgG2b	+++ *	−	−	−	−	−	+++	+++	+++	**−**	**−**	**−**	+++
**2**	3-5-23	IgG2b	−	−	++	−	−	−	−	+++	+++	**−**	**−**	**−**	+++
**3**	11-21-22	IgG2a	−	−	+++	−	−	−	−	+++	+++	**−**	**−**	**−**	+++
**4**	18-18-5	IgG1	−	+	+++	+	+	++	++	+++	+++	**−**	**−**	**−**	+++
**5**	17-3-11	IgG2b	−	+	+++	+	+	++	++	+++	+++	**+**	**−**	**−**	+++
**6**	14-24-5	IgG2b	++	++	+++	+++	+++	++	++	+++	+++	**+++**	**−**	**−**	+++
**7**	21-12-10	IgG1	+	++	+++	++	++	+	+	++	+++	**++**	**−**	**−**	+
**8**	17-16-16	IgG2a	**−**	**−**	**−**	**−**	**−**	**−**	**−**	+++	+++	**−**	**−**	**−**	+++
**9**	2-20-20	IgG2b	**−**	**−**	**−**	**−**	**−**	**−**	**−**	+++	+++	**−**	**−**	**−**	+++
**10**	3-5-4	IgG2a	**−**	**−**	**−**	**−**	**−**	**−**	**−**	+++	+++	**−**	**−**	**−**	+++
**11**	3-7-9	IgG2a	**−**	**−**	**−**	**−**	**−**	**−**	**−**	+++	+++	**−**	**−**	**−**	+++
**12**	3-7-19	IgG2a	**−**	**−**	**−**	**−**	**−**	**−**	**−**	+++	+++	**−**	**−**	**−**	+++
**13**	3-9-18-7	IgG2a	**−**	**−**	**−**	**−**	**−**	**−**	**−**	+++	+++	**−**	**−**	**−**	+++
**14**	8-10-16	IgG2a	**−**	**−**	**−**	**−**	**−**	**−**	**−**	+++	+++	**−**	**−**	**−**	+++
**15**	8-13-19	IgG2a	**−**	**−**	**−**	**−**	**−**	**−**	**−**	+++	+++	**−**	**−**	**−**	+++
**16**	10-19-19	IgG2b	**−**	**−**	**−**	**−**	**−**	**−**	**−**	+++	+++	**−**	**−**	**−**	+++
**17**	11-8-3	IgG2b	**−**	**−**	**−**	**−**	**−**	**−**	**−**	+++	+++	**−**	**−**	**−**	+++
**18**	11-11-2-7	IgG2a	**−**	**−**	**−**	**−**	**−**	**−**	**−**	+++	+++	**−**	**−**	**−**	+++
**19**	17-3-7	IgG2a	**−**	**−**	**−**	**−**	**−**	**−**	**−**	+++	+++	**−**	**−**	**−**	+++
**20**	17-16-28	IgG2a	**−**	**−**	**−**	**−**	**−**	**−**	**−**	+++	+++	**−**	**−**	**−**	+++
**21**	19-17-20	IgG2b	**−**	**−**	**−**	**−**	**−**	**−**	**−**	+++	+++	**−**	**−**	**−**	+++
**22**	22-8-6	IgG2a	**−**	**−**	**−**	**−**	**−**	**−**	**−**	+++	+++	**−**	**−**	**−**	+++
**23**	9-15-3	IgG1	**−**	**−**	**−**	**−**	**−**	**−**	**−**	+++	+++	**−**	**−**	**−**	+++
**24**	13-9-19-7	IgG2a	**−**	**−**	**−**	**−**	**−**	**−**	**−**	+++	+++	**−**	**−**	**−**	+++
**25**	10-2-9	IgG2a	**−**	**−**	**−**	**−**	**−**	**−**	**−**	+++	+++	**−**	**−**	**−**	+++
**26**	10-3-17	IgG2a	**−**	**−**	**−**	**−**	**−**	**−**	**−**	+++	+++	**−**	**−**	**−**	+++
**27**	19-9-13	IgG2b	**−**	**−**	**−**	**−**	**−**	**−**	**−**	+++	+++	**−**	**−**	**−**	+++
**28**	13-13-1	IgG1	**−**	**−**	**−**	**−**	**−**	**−**	**−**	+++	+++	**−**	**−**	**−**	+++
**29**	14-16-4	IgG1	**−**	**−**	**−**	**−**	**−**	**−**	**−**	++	++	**−**	**−**	**−**	+++
**30**	13-7-1	IgG2b	**−**	**−**	**−**	**−**	**−**	**−**	**−**	+	+++	**−**	**−**	**−**	+++
**31**	21-23-7	IgG1	**−**	**−**	**−**	**−**	**−**	**−**	**−**	+++	+++	**−**	**−**	**−**	+++
**32**	21-12-12	IgG1	**−**	**−**	**−**	**−**	**−**	**−**	**−**	++	+++	**−**	**−**	**−**	+++
**33**	2-13-3	IgG1	**−**	**−**	**−**	**−**	**−**	**−**	**−**	++	+++	**−**	**−**	**−**	+++
**34**	6-19-13	IgG1	**−**	**−**	**−**	**−**	**−**	**−**	**−**	++	+++	**−**	**−**	**−**	+++
**35**	7-20-1	IgG2a	**−**	**−**	**−**	**−**	**−**	**−**	**−**	+++	+++	**−**	**−**	**−**	+++
**36**	11-13-25	IgG2a	**−**	**−**	**−**	**−**	**−**	**−**	**−**	+++	+++	**−**	**−**	**−**	+++
**37**	13-18-2	IgG2a	**−**	**−**	**−**	**−**	**−**	**−**	**−**	+++	+++	**−**	**−**	**−**	+++
**38**	14-8-23	IgG2a	**−**	**−**	**−**	**−**	**−**	**−**	**−**	+++	+++	**−**	**−**	**−**	+++
**39**	16-21-32	IgG1	**−**	**−**	**−**	**−**	**−**	**−**	**−**	+++	+++	**−**	**−**	**−**	+++
**40**	22-3-9	IgG1	**−**	**−**	**−**	**−**	**−**	**−**	**−**	+++	+++	**−**	**−**	**−**	+++
**41**	13-13-7	IgG1	**−**	**−**	**−**	**−**	**−**	**−**	**−**	++	+++	**−**	**−**	**−**	+++
**42**	13-13-10	IgG1	**−**	**−**	**−**	**−**	**−**	**−**	**−**	+++	+++	**−**	**−**	**−**	+++
**43**	9-9-3	IgG1	**−**	**−**	**−**	**−**	**−**	**−**	**−**	++	+++	**−**	**−**	**−**	+++
**44**	8-18-15	IgG2a	**−**	**−**	**−**	**−**	**−**	**−**	**−**	+++	+++	**−**	**−**	**−**	+++
**45**	15-22-1	IgG1	**−**	**−**	**−**	**−**	**−**	**−**	**−**	+++	+++	**−**	**−**	**−**	+++
**46**	12-16-16	IgG1	**−**	**−**	**−**	**−**	**−**	**−**	**−**	++	++	**−**	**−**	**−**	+++

Recombinant HA proteins were derived from ^a^ A/California/07/2009 (H1N1pdm09), ^b^ A/Canada/720/2005 (H2N2), ^c^ A/Perth/16/2009 (H3N2), ^d^ A/Indonesia/5/2005 (H5N1), ^e^ A/Egypt/N05056/2009 (H5N1), ^f^ A/northern shoveler/Califormia/HKWF115/2007 (H6N1), ^g^ A/ruddy turnstione/NewJersey/563/2006 (H7N2), ^h^ A/Netherlands/219/2003 (H7N7), ^i^ Anhui/1 (H7N9), ^j^ A/Hong Kong/35820/2009 (H9N2), ^k^ B/Malaysia/2506/2004 (Victoria), and ^l^ B/Florida/4/2006 (Yamagata); ^m^ Purified Anhui/1 (H7N9) virus was used as the antigen.; * Reactivity of each mAb (1 μg/mL) was stratified according to the optical density at 450 nm, +++ (>1.0), ++ (0.5–1.0), + (0.1–0.5), and − (< 0.1).

**Table 2:** Hemagglutination inhibition (HI) and neutralization values (μg/mL) of the mouse mAbs against Anhui/1.

#	Clone	HI	Neutralization
**1**	7-20-10	>50	>50
**2**	3-5-23	>50	>50
**3**	11-21-22	50	>50
**4**	18-18-5	>50	>50
**5**	17-3-11	>50	>50
**6**	14-24-5	>50	>50
**7**	21-12-10	>50	>50
**8**	17-16-16	0.78	1.10
**9**	2-20-20	0.39	0.70
**10**	3-5-4	0.78	0.62
**11**	3-7-9	0.78	1.10
**12**	3-7-19	0.78	0.78
**13**	3-9-18-7	0.78	1.24
**14**	8-10-16	0.78	1.10
**15**	8-13-19	1.56	4.12
**16**	10-19-19	0.78	0.98
**17**	11-8-3	1.56	2.21
**18**	11-11-2-7	1.56	1.10
**19**	17-3-7	1.56	1.10
**20**	17-16-28	1.56	1.24
**21**	19-17-20	0.78	0.62
**22**	22-8-6	0.78	1.10
**23**	9-15-3	1.56	3.13
**24**	13-9-19-7	12.50	4.42
**25**	10-2-9	3.13	8.84
**26**	10-3-17	6.25	4.42
**27**	19-9-13	3.13	7.87
**28**	13-13-1	>50	>50
**29**	14-16-4	>50	>50
**30**	13-7-1	>50	>50
**31**	21-23-7	>50	>50
**32**	21-12-12	>50	>50
**33**	2-13-3	>50	>50
**34**	6-19-13	>50	>50
**35**	7-20-1	>50	>50
**36**	11-13-25	>50	>50
**37**	13-18-2	>50	>50
**38**	14-8-23	>50	>50
**39**	16-21-32	>50	>50
**40**	22-3-9	>50	>50
**41**	13-13-7	>50	>50
**42**	13-13-10	>50	>50
**43**	9-9-3	>50	>50
**44**	8-18-15	>50	>50
**45**	15-22-1	>50	>50
**46**	12-16-16	>50	>50

**Table 3:** Amino acid substitutions in the HA of Anhui/1 propagated in the presence of mAbs.

		Amino Acid Residue at the Indicated Position ^a^							
		60	78	83	135	144	205	226	505
**#**	Wild-type	D	Q	S	A	G	G	L	V
**8**	17-16-16	– ^b^	–	–	–	E	–	–	A
**9**	2-20-20	–	–	–	–	E	–	–	A
**10**	3-5-4	–	–	–	–	E	–	–	A
**11**	3-7-9	–	–	–	–	E	–	–	A
**12**	3-7-19	–	–	–	–	E	–	–	A
**13**	3-9-18-7	–	–	–	–	E	–	–	–
**14**	8-10-16	–	–	–	–	E	–	–	–
**15**	8-13-19	–	–	–	–	E	–	–	–
**16**	10-19-19	–	–	–	–	E	–	–	–
**17**	11-8-3	–	–	–	–	E	–	–	A
**18**	11-11-2-7	–	–	–	–	E	–	–	A
**19**	17-3-7	–	–	–	–	E	–	–	–
**20**	17-16-28	–	–	–	–	E	–	–	A
**21**	19-17-20	–	–	–	–	E	–	–	A
**22**	22-8-6	–	–	–	–	E	–	–	A
**23**	9-15-3	–	–	–	–	E	–	–	A
**24**	13-9-19-7	Y	–	–	T	–	–	Q	–
**25**	10-2-9	–	R	–	T	–	–	Q	–
**26**	10-3-17	–	H	–	T	–	E	–	–
**27**	19-9-13	–	–	P	T	–	–	Q	–

^a^ H3 Numbering; ^b^ Identical residues to the wild-type sequence.

**Table 4:** Neutralization values (μg/mL) of the mouse mAbs against mutant viruses.

#	Clone	Wild-Type	HA-G144E/V505A	HA-G144E	HA-A135T/L226Q	HA-A135T	HA-L226Q
**8**	17-16-16	1.24	>50	–	–	–	–
**9**	2-20-20	1.56	>50	–	–	–	–
**10**	3-5-4	1.24	>50	–	–	–	–
**11**	3-7-9	2.21	>50	–	–	–	–
**12**	3-7-19	1.97	>50	>50	4.42	–	–
**17**	11-8-3	4.42	>50	>50	9.92	–	–
**18**	11-11-2-7	2.21	>50	–	–	–	–
**20**	17-16-28	2.21	>50	–	–	–	–
**21**	19-17-20	2.21	>50	–	–	–	–
**22**	22-8-6	2.21	>50	–	–	–	–
**23**	9-15-3	3.94	>50	–	–	–	–
**24**	13-9-19-7	4.42	3.94	4.96	22.3	6.25	3.96
**25**	10-2-9	8.84	– ^a^	–	>50	6.25	2.21
**26**	10-3-17	8.84	–	–	>50	4.96	2.21
**27**	19-9-13	4.42	3.94	9.92	>50	21.0	8.84

^a^ Not tested.

**Table 5:** Neutralization values (μg/mL) of the mouse mAbs against H7N9 viruses.

#	Clone	Cluster I						Cluster II					Cluster III	Cluster IV		Cluster V
		AH/13 ^a^	HZ/13 ^b^	ST/14 ^c^	GD/14 ^d^	ZJ/14 ^e^	AH/14 ^f^	FJ/16 ^g^	HK/16 ^h^	HK/17 ^i^	HN/17 ^j^	ZJ/16 ^k^	ZJ6/17 ^l^	AH/16 ^m^	ZJ2/17 ^n^	TW/17 ^o^
**8**	17-16-16	1.1	1.2	1.1	2.2	2.5	1.2	1.0	1.2	5.0	4.4	6.3	7.9	9.9	6.3	4.4
**9**	2-20-20	1.6	1.6	2.0	2.2	5.0	1.8	1.1	1.2	4.4	8.8	4.8	8.8	9.9	9.9	14.7
**10**	3-5-4	1.1	1.1	1.1	2.2	2.2	1.1	1.0	1.2	3.9	4.4	4.4	4.4	5.0	4.4	3.9
**11**	3-7-9	1.1	1.2	1.1	2.2	2.2	1.8	0.9	1.2	4.4	6.3	5.0	7.9	12.5	5.0	4.4
**12**	3-7-19	1.1	1.1	1.1	2.2	2.2	1.2	0.6	1.1	4.4	4.4	3.9	4.8	7.9	4.4	3.9
**13**	3-9-18-7	3.1	2.0	2.0	2.2	3.1	2.0	1.1	2.0	4.4	5.6	5.0	8.8	7.9	6.3	2.5
**14**	8-10-16	3.1	2.2	2.2	3.1	4.4	2.2	1.1	2.2	5.0	4.4	5.0	5.0	6.3	5.0	4.4
**15**	8-13-19	4.4	4.4	4.4	5.3	8.8	5.0	2.2	4.4	5.0	15.8	8.8	9.9	17.7	9.9	8.8
**16**	10-19-19	1.2	2.0	1.6	2.2	4.4	2.2	1.1	1.1	3.9	7.9	4.4	8.8	8.8	8.8	15.8
**17**	11-8-3	2.5	3.1	4.4	4.4	4.4	2.2	2.2	2.2	5.0	5.0	4.4	8.8	9.9	8.8	9.9
**18**	11-11-2-7	2.2	2.5	2.2	4.4	4.4	4.4	1.2	2.2	4.4	7.9	5.0	7.3	9.9	6.3	4.4
**19**	17-3-7	1.1	2.5	2.2	3.1	2.4	2.2	1.1	0.8	5.0	8.8	4.4	14.0	9.9	5.6	4.4
**20**	17-16-28	2.0	1.2	2.0	2.5	2.2	2.2	1.1	1.1	4.4	5.0	4.4	9.9	5.6	5.0	3.9
**21**	19-17-20	1.6	2.0	1.6	2.5	2.5	2.0	1.1	1.1	4.4	5.6	6.3	8.8	17.7	8.8	17.7
**22**	22-8-6	1.1	1.1	1.2	2.2	2.2	2.0	0.8	1.1	4.4	4.4	4.4	5.0	5.0	5.6	3.1
**23**	9-15-3	8.8	2.2	2.5	4.4	4.4	3.1	1.6	2.2	4.4	8.8	4.4	8.8	8.8	9.9	5.0
**24**	13-9-19-7	8.8	8.8	8.8	15.8	25.0	6.3	6.3	8.8	>50	>50	19.8	29.3	31.5	17.7	>50
**25**	10-2-9	8.8	7.9	7.9	17.7	39.7	6.3	4.4	4.4	>50	>50	35.4	50.0	>50	>50	>50
**26**	10-3-17	8.8	8.8	8.8	19.8	50.0	8.8	4.4	5.0	>50	>50	39.7	>50	>50	>50	>50
**27**	19-9-13	8.8	8.8	9.9	35.4	>50	8.8	14.7	17.7	>50	>50	17.7	>50	>50	>50	>50

Viruses possessing HA derived from ^a^ A/Anhui/1/2013, ^b^ A/Huzhou/1/2013, ^c^ A/Shantou/1001/2014, ^d^ A/Guangdong/0048/2014, ^e^ A/Zhejiang/22/2014, ^f^ A/Anhui/09186/2014, ^g^ A/Fujian/1/2016, ^h^ A/Hong Kong/VB16049808/2016, ^i^ A/Hong Kong/214/2017, ^j^ A/Hunan/02287/2017, ^k^ A/Zhejiang/15/2016, ^l^ A/Zhejiang/6/2017, ^m^ A/Anhui/60928/2016, ^n^ A/Zhejiang/2/2017, or ^o^ A/Taiwan/1/2017 were used in this experiment.
